# How Does Social Class Affect Need for Structure during the COVID-19 Pandemic? A Moderated Mediating Model Analysis

**DOI:** 10.3390/ijerph19020932

**Published:** 2022-01-14

**Authors:** Ting-Ting Rao, Shen-Long Yang, Xiaowen Zhu

**Affiliations:** School of Humanities and Social Science, Xi’an Jiaotong University, Xi’an 710049, China; raotingtingpsy@stu.xjtu.edu.cn (T.-T.R.); yangsl@mail.xjtu.edu.cn (S.-L.Y.)

**Keywords:** need for structure, compensatory control, social class, COVID-19

## Abstract

The COVID-19 pandemic is profoundly affecting the minds and behaviors of people worldwide. This study investigated the differences in the need for structure among people from different social classes and the psychological mechanisms underlying this need, as well as the moderating effect of the threat posed by the pandemic. Using data collected from non-student adults in China, we found that the lower an individual’s social class, the lower their need for structure, and this effect was based on the mediating role of perceived control. However, the mediating effect was moderated by pandemic threat, and the above relationship existed only when this threat was low. When the level of pandemic threat was higher, neither the effect of social class nor of perceived control on the need for structure were significant. Specifically, in higher-threat situations, the need for structure among individuals from higher social classes and who had a higher sense of control increased significantly, meaning the mediating effect was no longer significant. This finding showed that under the threat of a pandemic, individuals who have a lower need for structure will still pursue and prefer structure and order. The theoretical and practical implications of the research are also discussed.

## 1. Introduction

Since the outbreak of the COVID-19 pandemic, our social culture and way of life have undergone profound changes [1,2]. Consequently, people are unknowingly experiencing the effects of this pandemic in their psychology and behavior. Many researchers have focused on the impact of COVID-19 on individuals’ subjective feelings and objective life [3]. However, underlying these different psychological performances, there may be some common psychological basis worth exploring.

The need for structure, a basic need for human beings [4], may be one such basic psychological variable that deserves our attention. It refers to the psychological need to perceive one’s existence and surroundings as clear, orderly, and predictable and not ambiguous or random [5], and could be the common inner psychological basis of many people’s external manifestations. For example, the need for structure is concretely embodied in our desire for clarity from the obscure events, hope to find rules for our daily work, or our need to experience order in the products we purchase [6]. Researchers have found that the need for structure can predict conspiracy beliefs about important social events [7], preference for work [8], and people’s understanding of news [9]. Compensatory control theory demonstrates that affirming social or physical structure is a means to compensate for personal control in an uncertain situation [10]. People who lack personal control are motivated to seek structure, order, and certainty in various ways [11]. As such, this motivation could be the underlying process that explains much of the psychological and behavioral performance of individuals during the COVID-19 pandemic.

It is worth noting that there are individual differences in this tendency. Previous studies have found that higher class individuals have a strong sense of personal control; therefore, their need for structure is relatively lower [7]. However, against the background of an uncertain environment, individuals tend to experience difficulty in maintaining their perceptions of a structured world, as demonstrated through belief in conspiracy theories [12], appeal of a dominant leader [13], and collective action [14], which may reinforce the need for structure. Therefore, it is worth exploring whether the level of the need for structure among individuals from different social classes has changed amidst the pandemic, and what is the mediating mechanism underlying this change. This will not only help us better understand the series of stress-related responses observed during the pandemic but also provide suggestions for pandemic prevention practice and management. Therefore, this study focused on the differences in the need for structure among individuals from higher and lower classes against the background of the COVID-19 pandemic, as well as the psychological mechanisms underlying this need.

### 1.1. Social Class, Perceived Control, and the Need for Structure

Studies focusing on the differences in the impact of the pandemic on individuals of higher and lower social classes in the USA [15] or in China [16,17] indicated that it imposed a significant negative impact on lower-class individuals. Moreover, previous research also found that perceived control positively affected individuals [18,19], while the need for structure negatively influenced them during the pandemic [20]. However, the relationship between social class, perceived control, and the need for structure is yet to be clarified. Subsequently, we explored their correlations from the existing literature (before the COVID-19 pandemic).

First, perceived control, the degree to which an individual feels that he or she is in control of the external world and not restricted by the environment [21], can negatively predict the need for structure. Research has shown that the lower an individual’s perceived control, the higher their need for structure [22]. Compensatory control theory provides an explanation for this effect: According to the theory [6,11], feeling a sense of control is a basic human need and provides an important guarantee for people to feel that the world and their objective environment is safe and orderly. However, people are often faced with situations that are beyond their control. To compensate for this lack of control, individuals’ needs for structure and order increase. Therefore, when confronted with incidents or uncertainty, individuals may upgrade their need for structure, with the lack of control as the psychological mediating factor. Many studies have supported this conclusion [23,24,25].

Second, social class, which refers to an individual’s material resources as well as their perceptions of rank comparing with others in society [26], can positively predict perceived control. Despite the objective indicators used to define social class in existing literature, in recent years researchers tended to depict individuals’ social class by integrating their perception of their own status in the social hierarchy with the traditional objective measurement. Therefore, several psychological studies on social class examined both the effect of objective class (some indices representing objective social status and material position, such as annual income, education level, and occupational reputation) and subjective class (a person’s subjective assessment of where they are on the social ladder) [27]. Previous research suggests that higher class individuals are more likely to have a higher sense of control, based on both objective and subjective social class indicators [21,28]. The cognitive theory of social class [26] attempts an explanation of this effect: The theory argues that people who belong to higher classes are more likely to enjoy more resources in their life, and their living environment can provide them with more protection; thus, it is easier for such individuals to pursue and achieve important life goals and follow their own desires freely. Conversely, due to the shortage of material resources in their life, individuals who belong to lower classes are often subjects of their environment and must consider more environmental factors and the influence of others in their social lives. In the long run, individuals belonging to higher classes develop a relatively higher sense of control, while those of lower classes tend to possess a low sense of control [26]. These findings have been consistently supported by different studies with East Asian [7] and American participants [29].

Based on these two aspects, we can infer a mediating relationship among social class, perceived control, and the need for structure. In particular, the higher the social class of the individual, the higher their perceived control, which further leads to a lower need for structure. Research has already found a mediation model of “social class → perceived control → need for structure” [7]; however, the study only took college students as its participants and only examined the effects of the subjective class, which rendered its results less compelling. In the present study, we retested this issue by using data obtained from adults in the context of the COVID-19 pandemic and examining the effects based both on objective and subjective social classes. We proposed the following hypothesis:

**Hypothesis** **1.** *Participants from lower social classes have lower levels of the need for structure than those from higher social classes, with perceived control playing a mediating role.*

### 1.2. Moderating Effect of the Threat of the Pandemic

The above hypothesis describes the general relationship among social class, perceived control, and the need for structure. However, considering the threat posed by the pandemic, we speculated that the relationship between the three variables will differ. Compensatory control theory demonstrates a lower sense of control leads individuals to seek structure, while also proposing some potential moderating variables that could remove the negative correlation between perceived control and the need for structure [6]. For example, individuals with lower perceived control indicated an increased preference for products that provide structure, but for individuals with a strong belief in God this effect was not significant [30]. The existing research on compensatory control theory has tended to focus on the conditions under which the need for structure will not increase among people with lower levels of perceived control [30,31]. Conversely, the present study examined whether the need for structure increases among people with a higher sense of control under the threat of a pandemic. Therefore, in addition to compensatory control theory, we introduced further theoretical perspectives to analyze this assumption.

The cognitive motivation model of stress [32] can provide a new perspective to investigate this issue. This theory focuses on the relationship between the stress experienced by individuals and their cognitive structure. Based on this model, the desire for certainty is one of the preconditions for individuals to construct cognitive structures in stressful situations. More importantly, the model suggests that when people feel stress and threat, their need for certainty increases. According to this view, we can conclude that in the context of a pandemic, people may feel pressure and threat, thus increasing their need for structure.

Further, there may be individual differences in the effects of this increase. Studies have shown that motivational threats most typically cause a specific motivation among people who have relatively lower general levels of that particular motivation [33,34,35]. That is, threat or stress from the environment may make the motivation salient for everyone, although it will have a stronger effect for those with low chronic motivation, thus causing them to become close to those that have high levels of that motivation. Therefore, if Hypothesis 1 holds true, people from higher social classes and with a higher sense of control will generally have a relatively lower need for structure. Therefore, we can further speculate that in a pandemic-threat situation, those individuals (from higher classes and/or with higher perceived control) are more likely to find their need for structure increases significantly than those from lower social classes and/or who have lower perceived control.

Based on the above analysis, we proposed the following three hypotheses regarding this moderating effect.

**Hypothesis** **2a.** *The threat of a pandemic can moderate the relationship between perceived control and the need for structure: When the level of pandemic threat is lower, perceived control negatively predicts the need for structure; when the threat level increases, the need for structure among individuals with higher perceived control will increase significantly, so that the predictive effect of perceived control on the need for structure will no longer be significant.*

**Hypothesis** **2b.** *The threat of a pandemic can moderate the relationship between social class and the need for structure: When the level of pandemic threat is lower, social class will negatively predict the need for structure; when the threat level increases, the need for structure among individuals from higher social classes will increase significantly, so that the predictive effect of social class on the need for structure will no longer be significant.*

**Figure 1:**
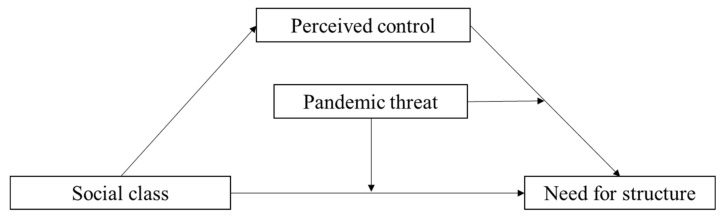
Hypothesized moderated mediating model.

**Hypothesis** **2c.** *The threat of a pandemic can moderate the mediating relationship of “social class → perceived control → need for structure” proposed in Hypothesis 1: When the level of pandemic threat is lower, the mediating effect will be significant; when the threat is higher, the mediating effect will not be established because the predictive effect of both social class and perceived control on the need for structure will no longer be significant (Figure 1).*

### 1.3. Overview of the Present Study

The present study examined the effects of both subjective and objective classes to test the above hypotheses to obtain more robust results. Data were obtained from a survey of non-student adults in China, and the hypothesized variable relationships and models were examined using a cross-sectional study design. Until 20 July 2021, the daily number of confirmed new COVID-19 cases in China had remained extremely low for many months. However, from late July to August, there were small outbreaks of COVID-19 in several Chinese provinces. According to official standards, several areas in China were classified as high risk or medium risk during this period. Within this context, we distributed questionnaires through an online platform to adults (excluding students) in various Chinese provinces in August 2021 to collect their self-reported scores on the above variables. We considered the official pandemic risk level (including high-risk, medium-risk, and low-risk areas) as the index of objective pandemic threat. Since residents in different regions of China faced different risk levels during this period, this Chinese sample was especially suitable for testing the present study’s hypotheses.

## 2. Materials and Methods

### 2.1. Participants

We recruited adult Chinese residents who were told this was a study on public social perceptions via Credamo (a Chinese questionnaire website, www.credamo.com (accessed on 10 January 2022). To thank them for their time, each participant who provided a valid response received ¥5. All participants were fully informed that their anonymity was assured, why the research was being conducted, and how their data would be used. As we aimed to test the effect of individuals’ social class, only non-student participants’ data were included. We included two questions to identify whether each participant’s data were valid, namely, “Please choose ‘strongly disagree’ for this question,” and “Please choose ‘not sure’ for this question,” which confirmed whether the participants had read the questions carefully. Data from participants who failed to answer these questions correctly were deleted. Students (according to the participants’ self-reported occupations) and participants who did not know the pandemic risk level in their area (according to the comparison between their self-reported risk level and the official risk level of the area where they lived) were also excluded (see also Section 2.2.5). In total, 92 participants with invalid data were excluded, leaving a final sample of 837 (43.8% male, *N* = 367, *M*_age_ = 31.93, *SD* = 6.82), which was higher than the recommended sample size (*N* ≈ 250) for obtaining stable coefficients based on the average effect size (r ≈ 0.20) in social and personality psychology [36,37].

### 2.2. Measures

#### 2.2.1. Objective Social Class

Three indicators of the objective social class (i.e., educational attainment, occupation, and monthly income) were measured. First, participants reported their education level by choosing one of the following six options: 1 = “primary school or below”, 2 = “junior high school”, 3 = “High school diploma or equivalent”, 4 = “junior college”, 5 = “bachelor’s degree”, or 6 = “postgraduate degree or higher”. Second, they reported their occupations in one of six categories, according to the classification criteria offered by previous Chinese research [38]: 1 = “student” (excluded); 2 = “temporary workers, unemployed people, unskilled workers, and agricultural workers, such as farmers”; 3 = “manual laborers, self-employed workers, skilled workers, and workers at the same level, such as industrial workers and service employees”; 4 = “general management personnel, general professional and technical personnel, and clerical staff, such as salespersons and drivers”; 5 = “middle management, middle-level professional and technical personnel, and assistant professional personnel, such as doctors, teachers, and engineers”; and 6 = “professional senior managers, senior professional and technical personnel, and professional supervisors, such as civil servants, company managers, and project managers.” Third, monthly income was divided into seven categories: <1000 RMB, 1000–2000 RMB, 2000–4000 RMB, 4000–8000 RMB, 8000–16,000 RMB, 16,000–32,000 RMB, and 32,000 RMB or more, with an overall value ranging from 1 to 7. Following the methods of previous studies [39,40], the three scores were then standardized, and an exploratory factor analysis extracted one principal component for the three items. The factor loading for each item was multiplied by the respective item score, and these scores were summed. Eigenvalues were then used to divide this sum and create the final objective class score. Higher scores represented a higher objective class.

#### 2.2.2. Subjective Social Class

The MacArthur Scale [41] was used to measure subjective social class. Participants were shown a 10-rung ladder and were asked to imagine that each level of the ladder represented different social classes in China (1 = the lowest class; 10 = the highest class). They were asked to consider their own social class and to choose a suitable number: a higher number indicated a participant’s higher perceived social class.

#### 2.2.3. Perceived Control

Perceived control was measured with a 12-item scale [21], which included items such as: “I can do just about anything I really set my mind to”, ”When I really want to do something, I usually find a way to succeed at it”, “Whether or not I am able to get what I want is in my own hands”, “What happens to me in the future mostly depends on me”, “Other people determine most of what I can and cannot do”, “There is little I can do to change many of the important things in my life”, “I often feel helpless in dealing with the problems of life”, “What happens in my life is often beyond my control”, “There are many things that interfere with what I want to do”, “I have little control over the things that happen to me”, “There is really no way I can solve all the problems I have” and “I sometimes feel I am being pushed around in my life” (the last eight items were reverse scored). These items were rated on a 7-point Likert scale (1 = strongly disagree; 7 = strongly agree). The responses were then averaged across the 12 items, with higher scores indicating a higher level of perceived control. Cronbach’s alpha in this study was 0.92.

#### 2.2.4. Need for Structure

The need for structure was measured with an 11-item Personal Need for Structure scale [42], which included items such as: “It upsets me to go into a situation without knowing what I can expect from it”, “I’m not bothered by things that interrupt my daily routine (reverse scored)”, “I enjoy having a clear and structured way of life”, “I like to have a place for everything and everything in its place”, “I find that a well-ordered life with regular hours makes my life tedious (reverse scored)”, “I don’t like situations that are uncertain”, “I hate to change my plans at the last minute”, “I hate to be with people who are unpredictable”, “I find that a consistent routine enables me to enjoy life more”, “I enjoy the exhilaration of being in unpredictable situations (reverse scored)”, and “I become uncomfortable when the rules in a situation are not clear”. These items were rated on a 6-point Likert scale (1 = strongly disagree; 6 = strongly agree). The responses were averaged across the 11 items, with higher scores indicating a higher need for structure. Cronbach’s alpha in this study was 0.89.

#### 2.2.5. Pandemic Threat

We used a relatively objective standard to measure the threat of the COVID-19 pandemic. As the Chinese government released risk levels for each region daily to reflect the threat of the pandemic in each area of China, the participants were asked to choose one of four options to indicate the pandemic risk level in their area. The higher the number they chose, the higher the threat of the COVID-19 pandemic in their location. After they made their choices, we compared their self-reported risk ratings of where they lived with the official risk level. If these two levels were inconsistent, the participant’s data were excluded (see also Section 2.1).

## 3. Results

### 3.1. Common Method Variance Test

This study not only used self-reported data, but also combined objective social class indicators and risk levels, which could help control for the effects of common methodological biases. Simultaneously, Harman’s single-factor test was used to examine the common method variance [43]. The result showed that the first factor accounted for 26.34% of the total variance and did not explain most of the variance (<40%). Thus, there was no obvious common methodological bias in this study.

### 3.2. Preliminary Analyses

The means, standard deviations, and correlation coefficients for main research variables are displayed in Table 1. The results indicated that both objective and subjective social class were positively correlated with perceived control, and those three variables above were all negatively correlated with the need for structure.

### 3.3. Mediating Effect of Perceived Control (Objective Social Class as Independent Variable)

**Figure 2:**
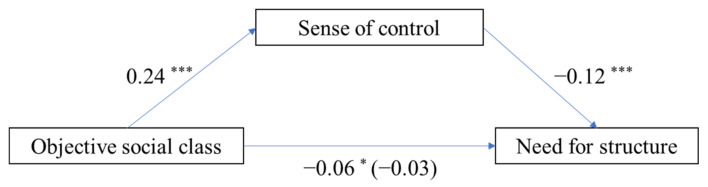
Model of the mediating role of perceived control in the association between objective social class and need for structure; * *p* < 0.05, *** *p* < 0.001.

Mediating effect analysis in PROCESS [44] was used to test the mediation effect using 1000 bootstrapped samples. Figure 2 displays the paths in the proposed model. Objective social class positively predicted perceived control (*b* = 0.24, *SE* = 0.03, *t* = 7.03, *p* < 0.001) and negatively predicted need for structure (*b* = −0.06, *SE* = 0.03, *t* = −2.18, *p* = 0.03). When we added objective social class and perceived control to the model simultaneously, perceived control negatively predicted need for structure (*b* = −0.12, *SE* = 0.03, *t* = −4.57, *p* < 0.001) and objective social class could not predict need for structure significantly (*b* = −0.03, *SE* = 0.03, *t* = −1.06, *p* = 0.29). Furthermore, bootstrapping analyses showed that perceived control mediated the pathway from objective social class to need for structure (indirect effect = −0.03, *SE* = 0.01, 95% CI = [−0.05, −0.02]), and the ratio of the indirect effect to total effect is 50.55%.

### 3.4. Moderated Mediating Effect of Pandemic Threat (Objective Social Class as Independent Variable)

We next tested for the moderating role of pandemic threat. Moderated mediating effect analysis in PROCESS [44] was used to test the moderated mediation effect using 1000 bootstrapped samples. Results (see Table 2) showed that objective social class was significantly associated with perceived control. More importantly, pandemic threat significantly moderated the impact of objective social class on need for structure and the impact of perceived control on need for structure. This suggests that the mediating effect among objective social class, perceived control, and need for structure was moderated by the pandemic threat. We further tested the conditional indirect effects. For a lower pandemic threat, the indirect effect of objective social class on the need for structure was significant (indirect effect = −0.08, 95% CI [−0.16, −0.01]), and for a higher pandemic threat, the effect was not significant (indirect effect = 0.03, 95% CI [−0.03,0.09]).

**Figure 3:**
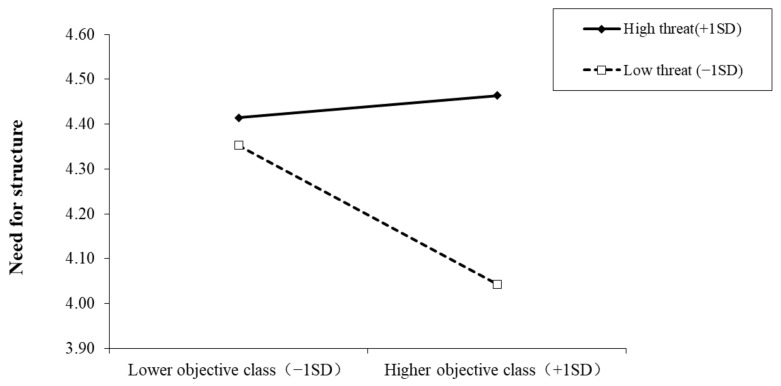
Interactive effect of pandemic threat and objective social class on need for structure. Note: Pandemic threat is graphed for two levels: high pandemic threat (1 *SD* above the mean) and low pandemic threat (1 *SD* below the mean).

We conducted simple slope tests to better understand the results regarding pandemic threat as a moderator. As depicted in Figure 3, when the pandemic threat was lower, the need for structure of the upper class individuals was significantly lower than that of lower class individuals (*b* = −0.15, *SE* = 0.04, *t* = −3.69, *p* < 0.001). However, when the pandemic threat was higher, this discrepancy disappeared (*b* = 0.02, *SE* = 0.03, *t* = 0.77, *p* = 0.44). Furthermore, for the lower objective class, pandemic threat could not predict the need for structure (*b* = 0.14, *SE* = 0.05, *t* = 0.97, *p* = 0.33), but for the higher objective class, the pandemic threat positively predicted the need for structure (*b* = 0.30, *SE* = 0.06, *t* = 5.05, *p* < 0.001).

**Figure 4:**
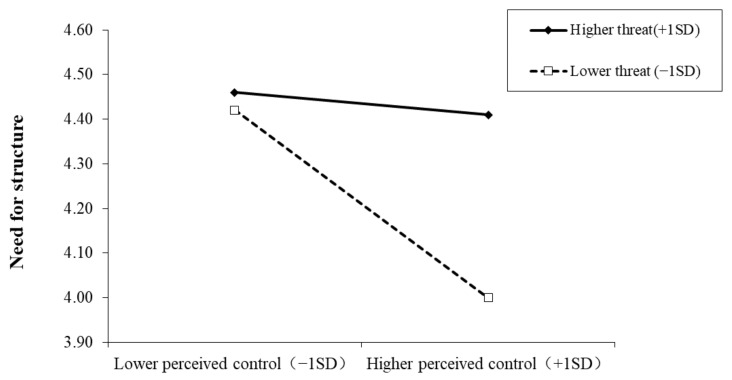
Interactive effect of pandemic threat and perceived control on need for structure. Note: Pandemic threat is graphed for two levels: higher pandemic threat (1 *SD* above the mean) and lower pandemic threat (1 *SD* below the mean).

Similarly, as depicted in Figure 4, when the pandemic threat was lower, people with higher perceived control reported a significantly lower need for structure than those with lower perceived control (*b* = −0.21, *SE* = 0.04, *t* = −5.03, *p* < 0.001). However, when the pandemic threat was higher, this discrepancy disappeared (*b* = −0.02, *SE* = 0.04, *t* = −0.61, *p* = 0.54). Furthermore, for individuals of lower perceived control, pandemic threat could not predict the need for structure (*b* = 0.03, *SE* = 0.05, *t* = 0.57, *p* = 0.57), but for those with higher perceived control, pandemic threat positively predicted the need for structure (*b* = 0.29, *SE* = 0.06, *t* = 5.21, *p* < 0.001).

### 3.5. Mediating Effect of Pandemic Threat (Subjective Class as Independent Variable)

**Figure 5:**
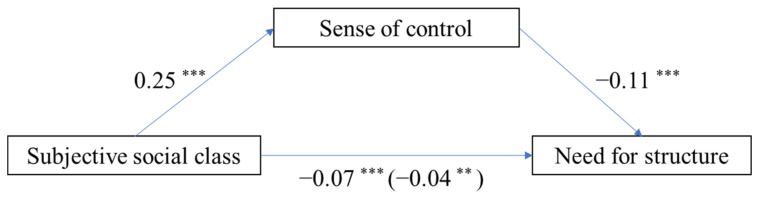
Model of the mediating role of perceived control in the association between subjective social class and need for structure; ** *p* < 0.01, *** *p* < 0.001.

Next, we used the subjective class as the independent variable, testing the same mediation model and moderated mediation model. Mediating effect analysis in PROCESS [44] was used to test the mediation effect using 1000 bootstrapped samples. Figure 5 displays the paths in the proposed model. Subjective social class positively predicted perceived control (*b* = 0.25, *SE* = 0.02, *t* = 10.82, *p* < 0.001) and negatively predicted need for structure (b = −0.07, *SE* = 0.02, *t* = −4.16, *p* < 0.001). When we added subjective social class and perceived control to the model simultaneously, perceived control negatively predicted need for structure (*b* = −0.11, *SE* = 0.03, *t* = −3.67, *p* < 0.001) and subjective social class significantly predicted need for structure (*b* = −0.04, *SE* = 0.02, *t* = −2.67, *p* = 0.008). Furthermore, bootstrapping analyses showed that perceived control mediated the pathway from subjective social class to need for structure (indirect effect = −0.03, *SE* = 0.01, 95% CI = [−0.04, −0.01]), and the ratio of the indirect effect to total effect was 37.07%.

### 3.6. Moderated Mediating Effect of Pandemic Threat (Subjective Social Class as Independent Variable)

We next tested for the moderating role of the pandemic threat. Moderated mediating effect analysis in PROCESS [44] was used to test the moderated mediation effect using 1000 bootstrapped samples. Results (see Table 3) showed that subjective social class was significantly associated with perceived control. More importantly, pandemic threat significantly moderated the impact of subjective social class on need for structure and the impact of perceived control on need for structure. This suggests that the mediating effect among subjective social class, perceived control, and need for structure was moderated by pandemic threat. We further tested the conditional indirect effects. For lower pandemic threat, the indirect effect of subjective social class on need for structure was significant (indirect effect = −0.05, 95% CI [−0.07, −0.03]), and for higher pandemic threat, the effect was not significant (indirect effect = 0.00, 95% CI [−0.02,0.01]).

**Figure 6:**
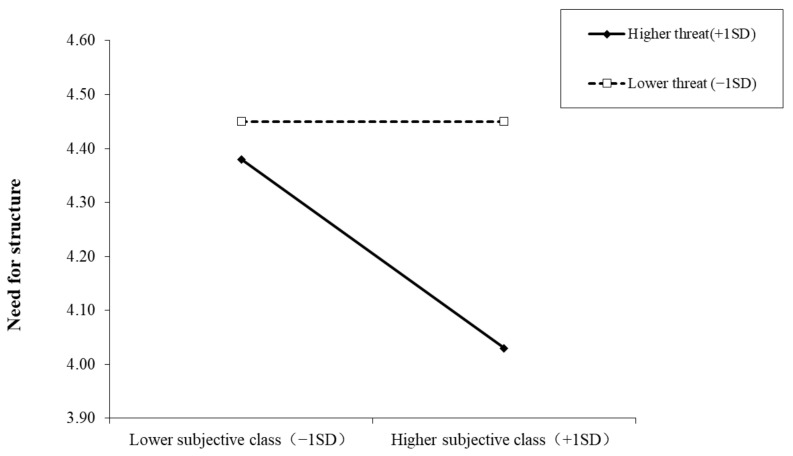
Interactive effect of pandemic threat and subjective social class on need for structure. Note: Pandemic threat is graphed for two levels: high pandemic threat (1 *SD* above the mean) and low pandemic threat (1 *SD* below the mean).

We conducted simple slope tests to better understand the results regarding pandemic threat as a moderator. As depicted in Figure 6, when the pandemic threat was lower, the need for structure of the upper-class individuals was significantly lower than that of the lower-class individuals (*b* = −0.13, *SE* = 0.03, *t* = −4.49, *p* < 0.001). However, when the pandemic threat was higher, this discrepancy disappeared (*b* = 0.00, *SE* = 0.02, *t* = −0.02, *p* = 0.98). Furthermore, for lower subjective class, pandemic threat could not predict the need for structure (*b =* 0.05, *SE* = 0.04, *t* = 1.12, *p* = 0.26), but for those with higher subjective class, pandemic threat positively predicted the need for structure (*b =* 0.30, *SE* = 0.06, *t* = 4.99, *p* < 0.001).

**Figure 7:**
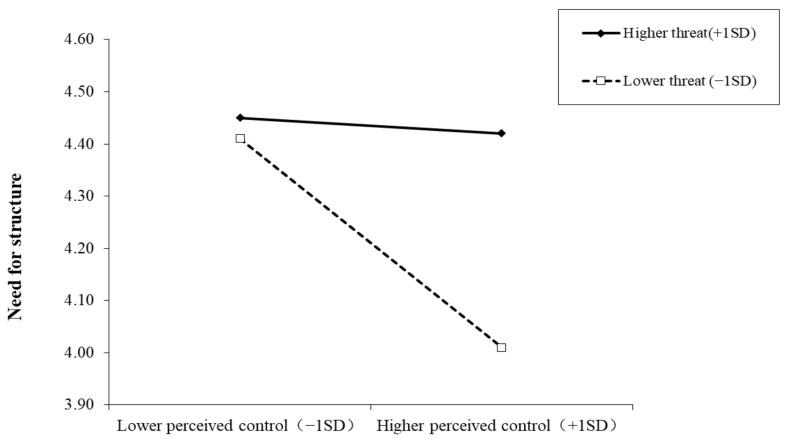
Interactive effect of pandemic threat and perceived control on need for structure. Note: Pandemic threat is graphed for two levels: higher pandemic threat (1 *SD* above the mean) and lower pandemic threat (1 *SD* below the mean).

Similarly, as depicted in Figure 7, when the pandemic threat was lower, people with higher perceived control had significantly lower need for structure than those with lower perceived control (*b =* −0.20, *SE* = 0.04, *t* = −4.61, *p* < 0.001). However, when the pandemic threat was higher, this discrepancy disappeared (*b =* −0.02, *SE* = 0.04, *t* = −0.45, *p* = 0.65). Furthermore, for individuals of lower perceived control, pandemic threat could not predict the need for structure (*b =* 0.03, *SE* = 0.05, *t* = 0.72, *p* = 0.47), but for those with higher perceived control, pandemic threat positively predicted the need for structure (*b =* 0.29, *SE* = 0.06, *t* = 5.13, *p* < 0.001).

Therefore, based on both the results of objective and subjective social classes, all the above hypotheses were supported by these data. Perceived control played a mediating role between social class and the need for structure, and pandemic threat moderated the mediating model.

## 4. Discussion

Based on the self-reported data in China during the COVID-19 pandemic and the objective risk level of each region given by official authority, this study tested the mediating effect and moderating effect hypotheses mentioned above. The results support all our hypotheses. Firstly, the results suggested that the mediating effect of perceived control on the relationship between social class (both objective and subjective) and need for structure is significant; thus, social class negatively predicted need for structure and this association was partially mediated by perceived control. Specifically, the lower an individual’s social class is, the lower his or her sense of control is, and, therefore, the higher his or her need for structure tends to be. Furthermore, the results also showed that pandemic threat moderated the relationship between perceived control and need for structure, and the relationship between social class and need for structure, finally leading to the moderated mediating effect. Under the condition of higher pandemic threat, individuals with higher perceived control increased their need for structure significantly, so the predictive effect of perceived control on need for structure was no longer significant. Similarly, under the condition of higher pandemic threat, individuals of higher social class increased their need for structure significantly, so to the predictive effect of social class on need for structure was no longer significant. Finally, the results showed the moderating effect of pandemic threat on the mediating model of “social class → perceived control → need for structure”. Therefore, all the hypotheses of this study were supported.

Previous studies tended to regard the need for structure as an independent variable and to examine its predictive effect on other psychological outcomes [45,46]. Conversely, few studies have taken the need for structure as a dependent variable and focused on the factors influencing it. Although the need for structure can be regarded as a relatively stable personality trait, it can also be influenced by other individual and environmental factors [47,48]. Especially in the context of uncertainty, need for structure can be regarded as the psychological basis of many psychological and behavioral factors, such as conspiracy theory thinking [49] and stereotyping [23]. It is meaningful to pay attention to need for structure and its influencing factors under the pandemic conditions. Accordingly, this study first examined the predictive effect of social class on need for structure, and found that people of lower class tend to develop a higher level of need for structure due to their relatively lower perceived control. This conclusion supports an expansion of previous research. Previous studies have found that social class positively predicts perceived control [21,29], while perceived control negatively predicts need for structure [30,31]. Although a previous study directly investigated the relationship among the three variables [7], it focused only on the student samples and the effect of subjective social class. The present study provides more solid evidence for this mediation model by using a sample of non-student adults from different provinces in China. This result more directly reveals the difference in need for structure among people of different social classes and the psychological mechanism underlining their lack of control. Compensatory control theory proposes that when personal control is threatened, individuals are more inclined to seek structure to compensate for personal control [6,11]. The results of this study show that people of lower class are more likely to feel the lack of control and then develop compensatory control, which has enlightenment value for the development of compensatory control research in the future.

In addition, this study’s most important finding is the moderating effect of pandemic threat. This phenomenon suggests that for individuals who generally lack one kind of motivation (e.g., the need for structure), it is more likely for them to be provoked by a threat (e.g., the COVID-19 pandemic) and the particular motivation of them will increases even more significantly. Similar views have been proposed and supported by previous studies [33,34,35] and the present study supplements the conclusions of this kind of research. At the same time, the results also support the cognitive motivation model of stress. This model suggests that an important aspect of the psychological impact of stress and threat is the increased desire for certainty [32], which is consistent with the conclusions regarding the need for structure made in the present study. We also found that, when faced with the threat of COVID-19, even those from higher classes (and who had a higher sense of control) experienced an increase in their need for structure, order, and certainty (though they did not in their normal state). This showed a cross-group consistency in psychological needs during the pandemic.

Why do individuals who normally deal better with uncertainties (individuals of higher class and with higher perceived control) experience the greater impact of the pandemic threat? We believe that it is necessary to distinguish their demonstration of the general state and the crisis state. In general, individuals of higher social class (and usually with a higher sense of control [21]) command more social resources, which can support them to cope with the challenges of normal life [26]. In contrast, lower-class individuals are less capable to deal with environmental threats due to a lack of resources, and, therefore, are more in need of certainty and order [7]. Under the condition of a new kind of threat (the COVID-19 pandemic), however, the upper-class individuals feel a threat that differs from the ones they face in their daily lives, which leads to a significant increase in their need for structure and avoidance of uncertainty. The threat of the pandemic has a lower impact on lower-class individuals, perhaps because they have been accustomed to threats from various domains. Therefore, instead of saying that the threat of the pandemic affects upper-class individuals more, it can also be interpreted as the unequal distribution of “normal” uncertainties and threats across different classes in daily life.

This study presents three theoretical implications that may provide some insight for the future research. First, the study observed that lower personal control is not the only source of the need for structure. On the contrary, people with a higher sense of control may also have a relatively high need for structure under certain conditions, such as the threat of a pandemic. Therefore, researchers of compensatory control theory need to further investigate the boundary conditions of the compensatory control model. Second, in terms of the need for structure, although this variable is usually regarded as a relatively stable personality trait [42], our study revealed that this basic need fluctuates under certain conditions. We found that the interaction of individual factors (social class, personal control) and environmental factors (pandemic threat) significantly predicted the need for structure. This suggests that future research on the need for structure should focus on the interaction effect between individual and environmental factors to comprehensively reveal the factors influencing this need. Third, this study adopted two operational definitions for the measurement of social class, that is, objective social class and subjective social class, and the effects were shown to be almost identical. Previous studies have found that the two have different effects on the prediction of some dependent variables [50]. For example, some studies found that subjective class positively predicts individual’s support for social system, while objective class is negatively correlated with system support [51,52]. In the present study, however, the effect of subjective class was almost the same as that of objective class, and together their effects supported all our assumptions. On the one hand, this reflects the stability of the conclusions of our study. On the other hand, it also shows that the concepts of subjective class and objective class still have a certain commonality and relevance.

In addition, this study also has some practical significance. The COVID-19 outbreak has greatly affected the way we think and live. At the same time, economic inequality, environmental problems, new technology, and many other factors have left the world in a state of uncertainty. The results of this study highlight that when faced with the threat and stress of a pandemic, people prefer to pursue a structured, orderly, and predictable life and do not want to face the random, uncontrollable, and changing physical and social environment. Moreover, even higher social class groups will have more needs and preferences for order and structure in the context of an epidemic or pandemic. Therefore, in the midst of the current pandemic, governments should consider whether their pandemic management policies meet the public’s need for structure and aim to maximize citizens’ sense of structure and order. Moreover, due to the consistency in the need for structure among the upper and lower social classes in the context of the pandemic, policy makers must also ensure the interests and security of both higher- and lower-class groups without distinction.

Finally, this study has some limitations, which should be investigated in future research to conduct a deeper exploration of the topic. First, the sample was derived only from China and was investigated in the context of small COVID-19 outbreaks in several Chinese provinces in July and August 2021. China’s pandemic-prevention policy is relatively stricter [17], and Chinese individuals exhibit higher levels of collectivism when facing the pandemic and the related policy measures [53]. Thus, Chinese individuals’ psychological response to COVID-19 may be influenced by certain unique sociocultural factors. Therefore, the behaviors of people in other countries and regions should be investigated in the future to test the conclusions of the present study. Second, since students and individuals who chose the wrong risk level of their residential area were excluded from this study, the representation of the present study findings may be slightly inadequate. Although this may not have affected the main conclusions of the present study, future research should include more representative samples. Third, our study’s conclusions rely on cross-sectional data, and the investigation of the relationship between variables was based on the correlation method, which cannot provide strong proof of a causal relationship. Consequently, alternative methods rooted in experimental design should be considered in future research to further verify the model and effects observed in this study, such as implicit-mediation analysis [54]. In addition, implementing longitudinal design is also a feasible way to test the robustness of the conclusions. Fourth, the present study only focused on the moderating effect of the threat of COVID-19. However, threats come from many sources in real life. Do all threats result in this effect? This question should also be examined in more complex studies in the future. Finally, the measurement of the participants’ occupation may not accurately describe the reality rank of some participants. Although the problem may be minor, it needs to be acknowledged. Future research can explore more ways to assess an individual’s objective social class.

## 5. Conclusions

Given the multiple psychological and behavioral impacts of the COVID-19 pandemic, we must focus on the common psychological underpinnings behind the typical manifestations of these impacts. The need for structure is one such motivational factor and can predict many psychological and behavioral performances. Despite the general individual differences in the need for structure (to be specific, higher-class individuals exhibit lower need for structure), individuals will demonstrate the same higher preference for structure under the threat of COVID-19 pandemic, regardless of their social class. Therefore, when formulating pandemic-prevention policies, the governments should give more consideration to protect the needs of structure, order, and certainty of individuals from different social classes, races, and groups.

## Figures and Tables

**Table 1:** Descriptive statistics and correlations for all variables.

Variables	*M* (*SD*)	1	2	3	4
1.Objective social class	0.00 (1.00)	1			
2.Subjective social class	5.71 (1.41)	0.37 ***	1		
3.perceived control	4.98 (1.00)	0.24 ***	0.35 ***	1	
4.Pandemic threat	3.17 (0.70)	0.06	−0.04	−0.01	1
5.Need for Structure	4.32 (0.77)	−0.08 *	−0.13 ***	−0.17 ***	0.16 ***

Note: *N* = 837; * *p* < 0.05, *** *p* < 0.001; *M* = mean; *SD* = standard deviation.

**Table 2:** Multiple regression analyses of moderated mediation effect.

Predictors	Model 1 (Criterion = Need for Structure)		Model 2 (Criterion = Perceived Control)		Model 3 (Criterion = Need for Structure)	
	*b*	*t*	*b*	*t*	*b*	*t*
Objective social class	−0.07	−2.58 *	0.24	7.23 ***	−0.03	−1.23
Pandemic threat	0.17	4.81 ***			0.16	4.61 ***
Objective social class ×Pandemic threat	0.13	3.28 *			0.08	2.29 *
Perceived control					−0.12	−4.09 ***
Perceived control ×Pandemic threat					0.13	3.51 ***
*R* ^2^	0.04		0.06		0.08	
*F*	9.79 ***		52.25 ***		8.14 ***	

Note: * *p* < 0.05, *** *p* < 0.001.

**Table 3:** Multiple regression analyses of moderated mediation effect.

Predictors	Model 1 (Criterion = Need for Structure)		Model 2 (Criterion = Perceived Control)		Model 3 (Criterion = Need for Structure)	
	*b*	*t*	*b*	*t*	*b*	*t*
Subjective social class	−0.06	−3.82 **	0.25	10.82 ***	−0.03	−2.05 *
Pandemic threat	0.17	4.73 ***			0.16	−4.60 ***
Subjective social class ×Pandemic threat	0.09	3.52 **			0.06	2.64 **
Perceived control					−0.11	−3.63 ***
Perceived control ×Pandemic threat					0.13	3.40 ***
*R* ^2^	0.05		0.12		0.08	
*F*	12.45 ***		117.15 ***		8.96 ***	

Note: * *p* < 0.05, ** *p* < 0.01, *** *p* < 0.001.

## Data Availability

Data will be provided if requested to the authors.
